# 0921. Effect of norepinephrine on intestinal oxygenation during fluid resuscitation of hemorrhagic shock in mice

**DOI:** 10.1186/2197-425X-2-S1-O29

**Published:** 2014-09-26

**Authors:** A Harrois, N Baudry, E Vicaut, J Duranteau

**Affiliations:** Hôpital de Bicêtre, Département d'Anesthésie-Réanimation, Le Kremlin Bicêtre, France; Laboratoire Microcirculation, Bioénergétique, Inflammation, Insuffisance Circulatoire Aigue, Université Paris 7/Paris 11, Paris, France

## Introduction

Hemorrhagic shock resuscitation aims at maintaining tissue perfusion while waiting for hemorrhage control. Fluid resuscitation remains the first line therapy to improve tissue perfusion but a vasoconstrictor (most often norepinephrine) may be associated to stabilize the mean arterial pressure (MAP) level and avoid hemodilution while waiting for transfusion. However, the arteriolar vasoconstriction induced by norepinephrine could have deleterious effects on tissue perfusion during hypovolemia due to hemorrhage.

## Objectives

To evaluate the effects of norepinephrine administration on intestinal perfusion and oxygenation during hemorrhagic shock resuscitation in mice.

## Methods

Tracheotomised and ventilated Balb/c mice were submitted to a controlled hemorrhagic shock to reach a MAP level of 40 mmHg during sixty minutes. Mice groups differed from their resuscitation between the 60^th^ and the 120^th^ minute: a fluid resuscitated group (FR) with NaCl 0,9% to reach a MAP level of 60 mmHg and a group resuscitated with fluid and norepinephrine (FRNE) to reach a MAP level of 60 mmHg. Mice were then retransfused with their shed blood and an equal volume of Ringer Lactate and were observed between the 120^th^ and the 165^th^ minute. Intestinal microcirculation was observed by intravital microscopy. Intestinal mucosal PO_2_ level (PO_2muq_) was measured by phosphorescence quenching (OxyMicro probe) at jejunal mucosal level. A sham group (no hemorrhage, no resuscitation) was constituted. Data are presented as mean ± SEM. The group effect on microcirculatory and oxygenation parameters was analysed with ANOVA and Mann Whitney tests.

## Results

Hemorrhagic shock induced an alteration of the intestinal microcirculatory perfusion with a decrease in the fraction of perfused villi and a decrease in the villous red blood cells flux. A decrease of the PO_2muq_ from 33,9 ± 3 to 8,2 ± 1 mmHg (p < 0,01) and from 32,9 ± 2 to 10,3 ± 2 mmHg (p < 0,01) was observed during hemorrhage in FR and FRNE groups respectively. During hemorrhagic shock resuscitation, MAP goal was reached in FRNE group (59 ± 1 mmHg) but not in FR group (52 ± 2 mmHg). Fluid resuscitation amount was 67 ± 14 µL.g^-1^ and 176 ± 19 µL.g^-1^ in FRNE and FR groups respectively (p < 0.05). The alteration of intestinal microcirculatory perfusion was corrected in the same proportion in FRNE and FR groups. The PO_2muq_ recovered to its basal level during resuscitation in the FR group: 30 ± 3 mmHg and in the FRNE group: 33 ± 2 mmHg.

## Conclusions

In a mice model of uncontrolled hemorrhagic shock, a MAP directed resuscitation associating norepinephrine and fluid resuscitation decreased blood loss and fluid requirements compared to a MAP directed resuscitation with fluid without norepinephrine while preserving intestinal microcirculatory perfusion and oxygenation.

